# Regulation of T Cell Signaling and Immune Responses by PTPN22

**DOI:** 10.1080/10985549.2024.2378810

**Published:** 2024-07-22

**Authors:** Rebecca J. Brownlie, Robert J. Salmond

**Affiliations:** School of Medicine, University of Leeds, Leeds, UK

**Keywords:** T cell, protein tyrosine phosphatase, PTPN22, immunotherapy, autoimmunity

## Abstract

Protein tyrosine phosphatases (PTPs) play central roles in the regulation of cell signaling, organismal development, cellular differentiation and proliferation, and cancer. In the immune system, PTPs regulate the activation, differentiation and effector function of lymphocytes and myeloid cells whilst single-nucleotide polymorphisms (SNPs) in PTP-encoding genes have been identified as risk factors for the development of autoimmunity. In this review we describe the roles for PTP nonreceptor type 22 (PTPN22) in the regulation of T lymphocyte signaling and activation in autoimmunity, infection and cancer. We summarize recent progress in our understanding of the regulation of PTPN22 activity, the impact of autoimmune disease-associated *PTPN22* SNPs on T cell responses and describe approaches to harness PTPN22 as a target to improve T cell-based immunotherapies in cancer.

## Introduction

Reversible protein tyrosine phosphorylation is controlled by opposing families of kinases (PTKs) and phosphatases (PTPs) and is essential for the regulation of gene expression, cellular growth, proliferation and division, and apoptosis. During immune responses, T cell antigen receptor (TCR), co-stimulatory CD28 and cytokine-dependent signaling pathways are propagated by PTKs and lead to the activation and proliferation of naïve T cells, and their subsequent differentiation into effector cell populations.^1–3^ To balance these activatory signals, inhibitory PTPs act to limit T cell activation and maintain immune homeostasis. Redundant and nonredundant roles for several nonreceptor type PTPs (PTPNs) in the regulation of T cell signaling, autoimmunity and anti-cancer immune responses have been determined.^4–9^ In this mini-review, we describe the roles of PTPN22 in these processes with a focus on the most recent studies in this area.

## Roles for PTPN22 in T Cell Signaling Pathways

PTPN22 is an ∼90 kDa protein that comprises an N-terminal catalytic domain, an interdomain region and a C-terminus that has four proline enriched regions, termed P1-P4. Human and murine PTPN22, termed lymphoid phosphatase (Lyp) and proline-, glutamic acid-, serine- and threonine enriched sequence (PEST)-enriched phosphatase (PEP) in some literature, share 70% sequence identity.^10^^,^^11^ PTPN22 is predominantly expressed in the cytoplasm of cells of hematopoietic origin where it interacts with the src homology 3 (SH3) domain of the inhibitory C-terminal Src kinase (CSK) through the P1 region.^12^^,^^13^ Other potential binding partners for PTPN22 in T cells include growth factor receptor bound protein 2 (Grb2),^14^ ubiquitin-associated and SH3 domain containing protein A (UBASH3A),^15^ end-binding protein 1 (EB1),^16^ proline-serine-threonine phosphatase interacting protein 1 (PSTPIP1)^17^ and 14-3-3 tau.^18^

Early studies showed that overexpression of PTPN22 in T cell lines inhibited TCR-induced cytokine production whilst substrate-trapping experiments suggested that tyrosine residues in the TCR zeta chain, Lck and zeta-associated protein of 70 kDa (ZAP70) kinases were direct substrates,^19–21^ implying that PTPN22 acts as a negative regulator of TCR signaling. More recent analysis has determined that PTPN22 expression is important for the re-recruitment of membrane-proximal CSK-containing nanoclusters that downregulate TCR signaling in late immunological synapses^22^ while in the absence of PTPN22, Lck phosphorylation and TCR-induced activation of downstream mitogen activated protein kinase (MAPK) and mechanistic target of rapamycin (mTOR) signaling is elevated.^23–26^

In addition to acting as a negative regulator of proximal TCR signaling, PTPN22 controls TCR-induced “inside-out” signaling via the Rap1 GTPase that regulates leukocyte function-associated antigen (LFA)-1-dependent adhesion.^24^^,^^25^ Thus, in the absence of PTPN22, the formation of LFA-1-dependent T cell-antigen-presenting cell (APC) conjugates is elevated.^25^ PTPN22 also acts as a direct negative regulator of LFA-1-dependent signaling,^27–29^ such that, in the absence of PTPN22, the velocity of migrating T cells and accumulation of LFA-1 and phosphorylated focal adhesion kinase (FAK) and Lck in the T cell leading edge is increased.^28^ Furthermore, recent studies have shown that PTPN22 may be recruited to and inhibit signaling downstream of the Type 1 interferon receptor (IFN-R) in CD4^+^ T cells, an effect blocked by tumor necrosis factor (TNF) receptor associated factor (TRAF) 3.^30^ By contrast, Jofra et al., reported that PTPN22 may be required for Type I IFN-R signaling in P14 TCR transgenic CD8^+^ T cells,^31^ indicating that the effects of PTPN22 in this signaling pathway may be cell type- and context-dependent.

As well as these direct roles for PTPN22 in T cell signal transduction pathways, increased TCR-driven signals in PTPN22-deficient T cells have indirect effects on cytokine receptor signaling. Thus, PTPN22 does not directly influence transforming growth factor β-dependent signaling but instead elevated TCR-driven interleukin (IL)-2 secretion by PTPN22-deficient T cells overcomes the antiproliferative effects of TGFβ.^32^ Similarly, enhanced TCR-induced CD25/IL-2 receptor alpha expression in PTPN22-deficient T cells may influence the magnitude of IL-2-dependent signaling under conditions of chronic T cell stimulation.^33^ Together these studies have demonstrated that PTPN22 acts as a negative regulator of T cell activation and contributes to the integration of antigen, integrin and cytokine receptor signals via both direct and indirect effects.

**Figure 1.**
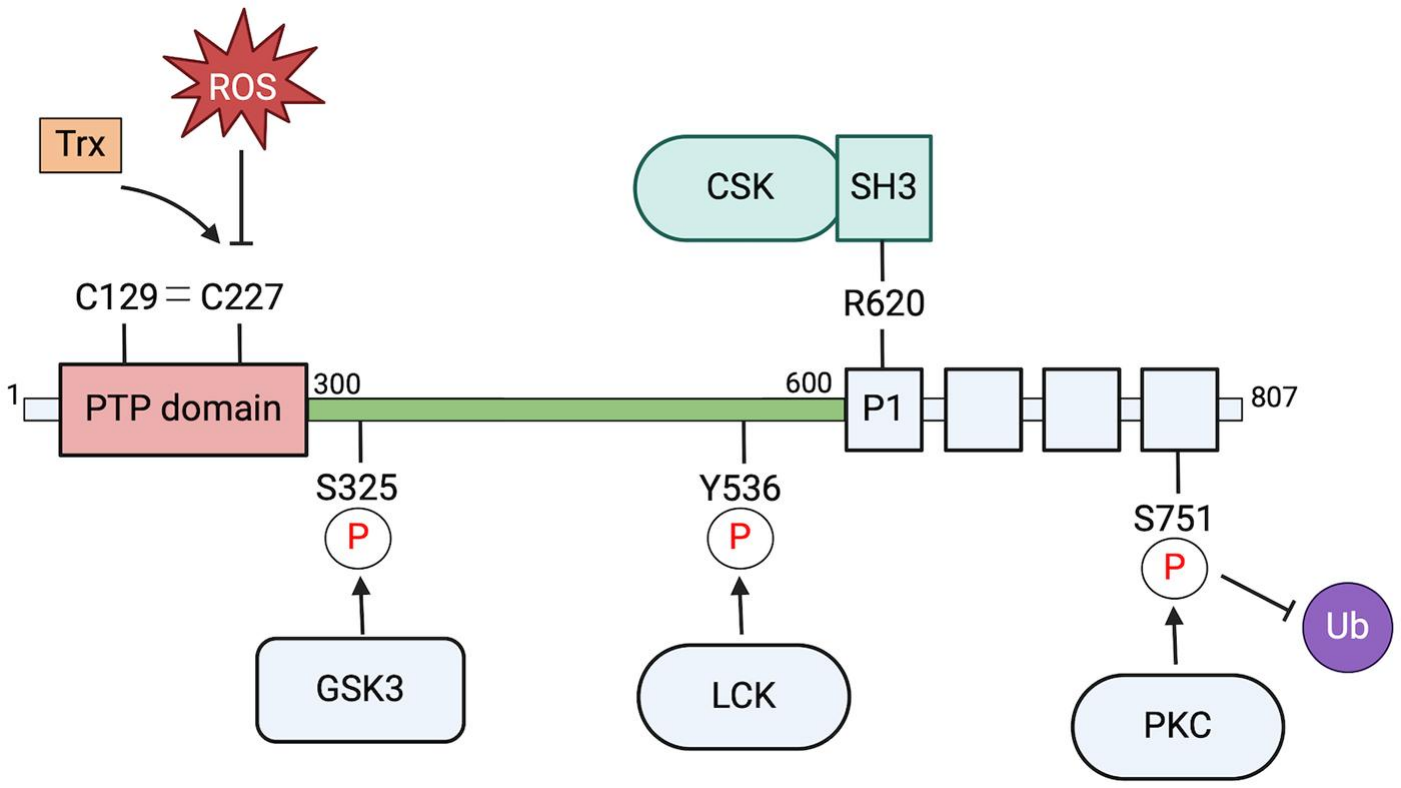
Regulation of PTPN22 activity. PTPN22 activity is regulated through post-translational modifications and protein-protein interactions. Residue C227 within the PTPN22 PTP domain is essential for catalytic activity and is subject to oxidation via reactive oxygen species (ROS) and reduction via thioredoxin (Trx). Formation of a disulphide linkage between C227 and noncatalytic C129 regulates these events. Interdomain region residue S325 is inducibly phosphorylated by GSK3 and residue Y536 by Lck, resulting in enhanced and reduced PTPN22 activity, respectively. Residue R620 in the P1 region is essential for PTPN22 association with the CSK SH3 domain and regulates PTPN22 activity and cellular localization. Residue S751 within the C-terminal region is phosphorylated by PKC which reduces ubiquitin (Ub)-dependent degradation of PTPN22.

A key question is: how is PTPN22 activity and function regulated in T cells? Association with CSK is an important factor in regulating PTPN22 subcellular localization and activity (Figure 1). Variants of PTPN22 that lack CSK binding (PTPN22 R620W) have elevated membrane localization^34^ and have greater phosphatase activity.^35^ Of note, association of PTPN22 and CSK is low in resting T cells but enhanced following TCR stimulation suggesting dynamic regulation of these negative regulatory proteins during T cell activation.^36^

PTPN22 is itself phosphorylated following TCR stimulation (Figure 1). Thus, protein kinase C-dependent phosphorylation of residue serine 751 (S751) prolongs PTPN22 protein half-life, by limiting its ubiquitinylation and proteosome-dependent degradation, and regulates membrane localization and association of PTPN22 with CSK.^37^ A phosphorylation-deficient PTPN22 S751A mutant protein was more prone to degradation and also demonstrated enhanced inhibitory activity due to altered cellular localization.^37^ PTPN22 is also phosphorylated at residue tyrosine 536 (Y536) by Lck following TCR stimulation.^38^ Mutation of Y536 results in gain-of-function activity, suggesting that phosphorylation of this residue has an inhibitory effect on PTPN22 phosphatase activity and serves to potentiate TCR signaling.^38^ Furthermore, PTPN22 R620W that lacks CSK binding capacity is less efficiently phosphorylated at Y536 by Lck.^38^ A recent study from the Bottini laboratory identified PTPN22 residue serine 325 (S325) as being inducibly phosphorylated by glycogen synthase kinase 3 (GSK3) following TCR stimulation.^39^ In this study, S325 was substituted for either an alanine residue (S325A) or glutamic acid residue (S325E) to block phosphorylation or mimic constitutive phosphorylation, respectively. In T cells expressing PTPN22 S325A, TCR-induced phosphorylation of PTPN22 substrates Lck and ZAP70 was elevated compared to control cells, suggesting loss of function, whereas the opposite was true in cells expressing PTPN22 S325E.^39^ These data suggest that TCR-driven, GSK3-dependent phosphorylation of S325 enhances PTPN22 function and subsequent downregulation of TCR signaling.

Similar to other PTP family members, PTPN22 catalytic activity is dependent upon an active site cysteine residue (C227) and is subject to redox regulation (Figure 1). The crystal structure of the PTPN22 catalytic domain indicates that C227 forms a disulphide bond with noncatalytic residue C129.^40^ Recent studies from the Holmdahl group showed that mutation of C129 (C129S) renders PTPN22 more susceptible to oxidative inactivation, less able to be reactivated by reduction through the thioredoxin pathway and reduces phosphatase activity.^41^

## Regulation of T Cell Homeostasis and Immune Responses by PTPN22

To decipher the role of PTPN22 in regulating the immune response, several research teams have generated *Ptpn22*-deficient or mutant mouse strains. Deletion of PTPN22 does not markedly impact on T cell developmental processes in the thymus either in the context of a polyclonal repertoire or in TCR transgenic models,^23–25^^,^^42^ although one study did report increased selection of thymic regulatory T cells (Treg) in *Ptpn22^-/-^* mice.^43^ More recently, a study showed that transgenic overexpression of a phosphatase-dead mutant PTPN22 in mice reduced thymic cellularity with minor effects on negative and positive selection of double-positive thymocytes.^44^ Initial analysis of T cell phenotypes in *Ptpn22^-/-^* mouse models demonstrated that in the absence of PTPN22, naïve T cell functions were comparable to wild-type T cells whereas effector T cell responses were enhanced leading to lymphadenopathy, splenomegaly and the accumulation of elevated numbers of effector/memory phenotype T cells and increased serum immunoglobulin (Ig).^23^^,^^24^ Follicular helper T cell numbers and functions are elevated in the absence of PTPN22, contributing to an increased number of splenic germinal centers and increased antibody levels.^45^ In addition, knock-in mice expressing a PTPN22 C129S protein had elevated CD4^+^ T helper 1 (Th1) cell responses and developed more severe disease in a glucose-6-phosphate isomerase peptide-induced model of inflammatory arthritis^41^ demonstrating the importance of this noncatalytic cysteine residue and suggesting a role for redox regulation of PTPN22 function in vivo.

These data are consistent with a predominantly inhibitory function for PTPN22 in T cells while the more marked impact of PTPN22-deficiency on effector T cells has been linked to higher PTPN22 expression levels in these subsets as compared to naïve T cells.^46^ Indeed, it is possible that upregulation of PTPN22 expression serves as a feedback mechanism to limit inflammatory effector T cell responses. However, experiments using *Ptpn22-*deficient mice crossed to a TCR transgenic background demonstrated that naïve T cell responses are also impacted by the absence of PTPN22. In this regard, when activated with a high-affinity ovalbumin-derived peptide ligand (pOVA), control and *Ptpn22*-deficient naïve CD8^+^ OT-I TCR transgenic T cell responses were comparable. However, in the absence of PTPN22, responses of naïve OT-I T cells to low affinity pOVA antigens were elevated, indicating that PTPN22 acts as a key determinant of TCR ligand discrimination.^25^ In effector and memory-phenotype CD8^+^ T cells, the absence of PTPN22 results in markedly enhanced inflammatory cytokine production and cytotoxic capacity, particularly in response to weak antigenic stimulation.^25^^,^^32^^,^^47^ Of note, earlier studies investigating *Ptpn22*-deficient mouse phenotypes predominantly used CD3/CD28-antibodies, which trigger a strong activating signal, for in vitro T cell experiments.^23^^,^^24^ Subsequent work has determined that clustered regularly interspaced short palindromic repeats (CRISPR)-Cas9-mediated deletion of *PTPN22* also predominantly impacts upon responses to low affinity antigen, with a more modest effect on responses to strong antigens, in human T cells.^26^^,^^48^

To define the underlying mechanisms underpinning the observed accumulation of effector-memory T cells in *Ptpn22^-/-^* mice, several studies have determined the impact of PTPN22 on lymphopenia-induced T cell proliferation. Adoptive transfer of either naïve TCR transgenic CD8^+^ T cells or polyclonal CD4^+^ T cells to lymphopenic *Rag1^-/-^* or nonobese diabetic (NOD) severe combined immunodeficiency (SCID) common-γ chain-deficient (NSG) mice or sublethally irradiated hosts demonstrated that proliferation of CD8^+^ and CD4^+^ *Ptpn22^-/-^* T cells was markedly enhanced in vivo compared to *Ptpn22^+/+^* counterparts.^25^^,^^31^^,^^46^ Similarly, following induction of lymphopenia by antibody-mediated T cell depletion, reconstitution of the T cell pool was accelerated in *Ptpn22^-/-^* as compared to control mice.^49^ Neutralization of IL-7, a known driver of T cell expansion in lymphopenic environments, did not prevent the increased proliferation of *Ptpn22^-/-^* T cells,^25^^,^^46^^,^^49^ indicating that TCR triggering by low affinity self- or environmental antigens was likely responsible for driving this phenotype.

Despite evidence for enhanced basal T cell activation and increased sensitivity to TCR stimulation in *Ptpn22^-/-^* mice, PTPN22-deficiency alone does not provoke spontaneous autoimmunity^23^^,^^24^ and has variable effects when crossed with other risk alleles and/or autoimmune-prone genetic backgrounds.^42^^,^^45^^,^^50–52^ In this regard, PTPN22-deficiency favors CD4^+^ Th1 differentiation and may subsequently limit Th17-dependent inflammation.^29^^,^^42^ Moreover, regulatory T cell (Treg) cell numbers and function are elevated in *Ptpn22^-/-^* mice^24^^,^^43^^,^^53^ suggesting that elevated inflammatory T cell activation is balanced by corresponding suppressive Treg activity. In this regard, naïve *Ptpn22^-/-^* CD4^+^ T cells more readily adopt a Treg phenotype under conditions of weak TCR stimulation in vitro, compared to control T cells,^54^ whilst *Ptpn22^-/-^*, but not *Ptpn22^+/+^*, Tregs are able to limit *Ptpn22^-/-^* effector T cell-mediated inflammation in an adoptive T cell transfer model of colitis.^24^

## Regulation of T Cell Self-Reactivity by PTPN22

Mechanisms to prevent T cell-mediated autoimmunity include the deletion of self-reactive thymocytes (central tolerance) and suppression of peripheral T cell reactivity via inhibitory cell types such as Tregs (peripheral tolerance). However, when these mechanisms are circumvented, inflammatory T cells can provoke organ-specific or systemic inflammation. Expression of specific human leukocyte antigen (HLA) haplotypes are the strongest genetic risk factors for autoimmunity, indicating the vital role for T cells in these diseases. The phenotype of CD4^+^ T cells responsible for provoking autoimmunity varies depending on the disease but can broadly be divided into either T helper 17 (Th17) cells, involved in diseases such as multiple sclerosis (MS), or Th1 cells implicated in diseases such as Type 1 diabetes (T1D). Of note, single nucleotide polymorphisms (SNPs) in *PTPN22* have been identified as risk factors for the development of autoimmune diseases.^55–59^ In particular, expression of a missense SNP (1858T) results in an amino acid substitution (R620W) within the P1 region that abrogates PTPN22 association with CSK. Expression of PTPN22 R620W is associated with increased risk of T1D,^60^ rheumatoid and juvenile idiopathic arthritis (RA/JIA),^61–64^ systemic lupus erythematosus (SLE),^65^ Grave’s disease^66^ and pernicious anemia,^67^ but not psoriasis^63^^,^^68^ or MS.^63^^,^^69^ By contrast, expression of PTPN22 R620W reduces the risk of developing Crohn’s disease^70^ whilst expression of a loss-of-function variant PTPN22 R263Q is associated with a reduced risk of developing RA and SLE.^71^^,^^72^ It is likely that effects on T cells,^73^^,^^74^ B cells,^75^^,^^76^ myeloid cells^77^^,^^78^ and granulocytes^79–81^ all contribute to autoimmunity phenotypes associated with expression of PTPN22 R620W. Autoimmune diseases associated with the production of autoantibodies have been linked to expression of the R620W variant, whereas those in which autoantibodies play less of a role have not.^55^

As described above, disease-associated PTPN22 R620W fails to bind CSK, has elevated phosphatase activity and altered cellular localization, implying that this SNP results in a gain-of-function mutation.^34^^,^^35^^,^^38^ Indeed, T cells from healthy individuals^73^^,^^82^ as well as T1D patients^74^ with homozygous expression of the *PTPN22* 1858T allele were reported to be hyporesponsive to TCR stimulation consistent with the notion that PTPN22 R620W is a gain-of-function variant that blocks T cell activation. The disease-associated allele may also impact on T cell development and/or homeostasis as suggested by elevated numbers of circulating Tregs in SLE^83^ and T1D^84^ patients expressing *PTPN22* 1858T. However, these studies could not rule out the possibility that T cell hyporesponsiveness associated with PTPN22 R620W expression was, at least in part, a consequence of chronic exposure to inflammation in vivo and/or secondary to effects of the variant protein on other immune cells’ function. Indeed, a recent study reported that overexpression of PTPN22 R620W had a lesser inhibitory effect on proximal TCR signaling in human CD4^+^ T cells compared to overexpression of the non-risk allele,^85^ suggesting that it may represent a loss-of-function variant. Furthermore, “knock in” mice expressing the disease-associated PTPN22 R619W variant (the mouse orthologue of human PTPN22 R620W) display a similar phenotype compared to *Ptpn22^-/-^* strains.^29^^,^^49^^,^^86–89^ Thus, PTPN22 R619W mice develop splenomegaly, associated with accumulation of activated effector T cell populations, increased germinal center formation and elevated serum Ig levels, similar to PTPN22-deficient mice. Importantly, PTPN22 R619W mice on a mixed genetic background develop autoantibodies and are more susceptible to drug-induced diabetes.^87^ Furthermore, Sherman et al., demonstrated that expression of the disease-associated R619W variant on the NOD genetic background results in earlier onset and penetrance of diabetes.^90^ Interestingly, whilst deletion of *Ptpn22* also enhances disease penetrance on the NOD background, the phenotype is more severe in the R619W knock in as compared to knockout strains, suggesting a more complex phenotype associated with expression of the disease-associated polymorphism.^90^ By contrast, and in accordance with human studies,^70^ expression of R619W protects against intestinal inflammation in an acute model of dextran sodium sulfate-induced colitis.^91^^,^^92^ Homozygous expression of PTPN22 R619W also influences negative and positive selection in the thymus suggesting that the T cell/TCR repertoire may be altered in these mice.^87^ However, in contrast to the human data,^73^^,^^74^^,^^82^ TCR signaling was elevated in mouse cells expressing PTPN22 R619W,^87^ suggesting differences between the effects of disease-associated PTPN22 variants in human and mouse T cells.

Recent studies from the Rawlings laboratory have begun to reconcile these apparent discrepancies. These researchers have used CRISPR-Cas9 approaches to engineer *PTPN22* knockout or R620W knock-in alleles in naïve human cord blood T cells.^48^ Results demonstrated that PTPN22 R620W expression phenocopied PTPN22 knockout in human T cells; in both cases signaling was elevated in response to low affinity, but not high affinity, TCR ligands compared to control T cells.^48^ Taken together with data from mouse studies,^25^ these observations suggest that a principle function of PTPN22 is to limit activation of weakly self-reactive T cells and that in the absence of PTPN22, or in the presence of the disease-associated R619W/R620W variant, increased TCR signaling and activation of such cells is permitted, contributing to induction of autoimmune disease. It should be emphasized that disease-associated *PTPN22* polymorphisms will affect the function of many leukocyte lineages in addition to T cells that may influence onset the autoimmunity.

## Role of PTPN22 in T Cell Responses to Infection

During immune responses to infection, T cells play an important role in clearing pathogens. CD4^+^ T cells can adopt specialized effector phenotypes (e.g., Th1, Th2, Th17) that orchestrate responses to varied pathogen types (e.g., viral, bacterial, fungal, protozoal and helminth pathogens) whilst CD8^+^ T cells directly kill pathogen-infected cells. Following clearance of a primary infection, the generation of long-lived memory T cells protects against re-infection. Following the identification of PTPN22 R620W as a risk factor for autoimmunity, several studies have assessed its association with immune responses to infection. Carriers of the 1858T allele were found to be more at risk of invasive pneumococcal disease within a UK patient cohort suggesting that expression of PTPN22 R620W also influences responses to bacterial infection.^93^ An early study suggested that expression of the 1858T allele may be protective against mycobacterial infection^94^ although a recent meta-analysis did not reproduce this finding.^95^ Furthermore, variable effects of expression of PTPN22 R620W on responses to influenza vaccination have been reported. Thus, one study reported that R620W expression was associated with impaired induction of flu-specific CD4^+^ T cells and antibody production following vaccination,^96^ while a second study suggested no such effect.^97^ Recent work identified PTPN22 itself as a target for the HIV Vpr protein during infection of primary human CD4^+^ T cells.^98^ However, the functional consequence of PTPN22 degradation in T cells during HIV infection has yet to be defined.

Researchers recently identified a link between expression of the autoimmune associated PTPN22 R620W variant with onset of myalgic encephalomyelitis/chronic fatigue syndrome (ME/CFS).^99^ Interestingly, this association was only found in patients that reported onset of symptoms following acute infection.^99^ These data suggest that altered immune responses to pathogens may be more likely to precipitate ME/CFS in individuals expressing *PTPN22* risk alleles. Further work is required to elucidate the mechanism by which expression of PTPN22 R620W pre-disposes to ME/CFS and to define whether this is a T cell-dependent process.

Recent work by the Sherman group assessed the impact of PTPN22 disease-associated alleles on antiviral responses in mice. These researchers reported that PTPN22 R619W knock in mice had enhanced capacity to clear chronic lymphocytic choriomeningitis virus (LCMV-clone 13) infection as compared PTPN22 wild-type (WT) controls.^100^ The improved control of LCMV infection was associated with increased numbers of antiviral effector CD4^+^ Th1 and cytotoxic CD8^+^ T cells in PTN22 R619W mice compared to controls. Furthermore, adoptive cell transfer of PTPN22 WT or R619W virus-specific TCR transgenic CD4^+^ to either PTPN22 WT or R619W mice demonstrated that the enhanced virus-driven accumulation of R619W Th1 cells was mediated by a combination of cell intrinsic and extrinsic factors.^100^ A previous study from the same group demonstrated that *Ptpn22^-/-^* mice also clear LCMV-Clone 13 infection more efficiently than *Ptpn22^+/+^* controls, with PTPN22-deficient CD4^+^ T cells retaining functional capacity and being less prone to exhaustion in chronic infection.^101^ Similar findings were reported by Jofra et al., although it was suggested that the lack of a T cell exhaustion phenotype in *Ptpn22^-/-^* mice may be a consequence of cell-extrinsic factors.^102^

## PTPN22 as a Target for Cancer Immunotherapy

In recent decades, the development and implementation of immunotherapies that boost T cell reactivity to tumors has revolutionized cancer treatment and patient outcomes. These therapies fall broadly into two main categories; (i) those that boost endogenous immune responses to tumors by blocking immunosuppressive immune checkpoint receptors such as programmed death-1 (PD-1) and cytotoxic T lymphocyte antigen (CTLA)-4, (ii) adoptive T cell therapies (ACT) in which autologous T cells are engineered to express tumor-reactive receptors (e.g., chimeric antigen-receptors (CARs)) ex vivo prior to reinfusion. Studies have suggested that targeting inhibitory PTPN family proteins, including PTPN22, may also serve to boost anti-cancer immune responses and may be used in conjunction with established immune checkpoint blockade and ACT approaches.^5^,^103^

**Figure 2.**
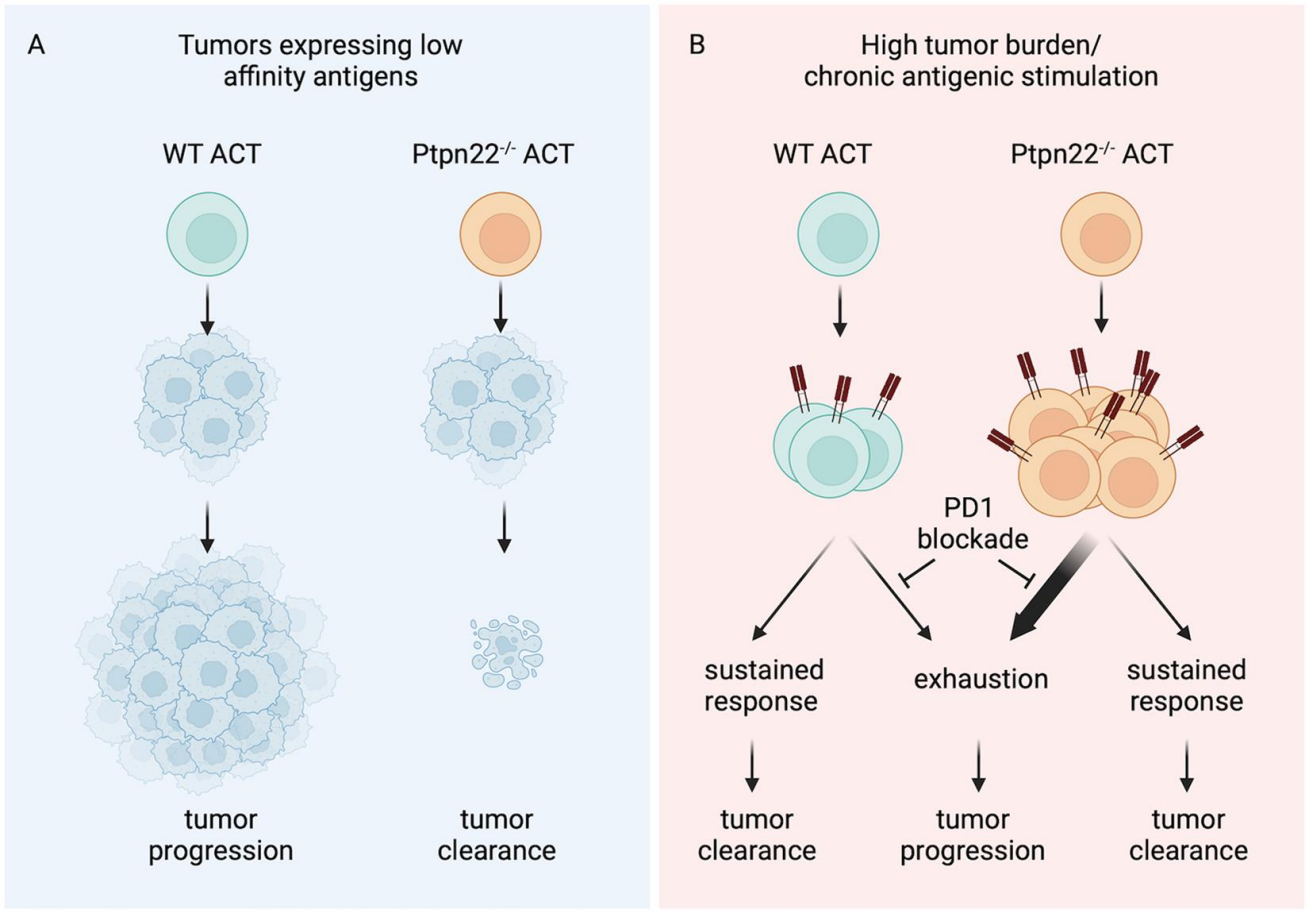
PTPN22 as a target to enhance T cell ACT responses in cancer. (A) Deletion of PTPN22 enhances the efficacy of T cell adoptive cell transfer (ACT) antitumor responses as compared to wild-type (WT) ACT, particularly in response to low affinity tumor-associated antigens. In tumor environments where adoptively transferred T cells are subjected to chronic antigenic stimulation (B), PTPN22-deficient T cells initially proliferate to a greater extent than control T cells but may be more prone to an exhausted phenotype, with high expression of immune checkpoint receptors. Blockade of receptors such as PD-1 may prevent functional exhaustion.

Studies have demonstrated that ACT using naïve or effector *Ptpn22^-/-^* OT-I TCR transgenic CD8^+^ T cells is superior to control OT-I ACT in mediating clearance of ID8 ovarian carcinoma or EL4 lymphoma cells expressing weakly antigenic pOVA as tumor-associated antigens^32^^,^^47^^,^^104^ (Figure 2). Furthermore, in vitro polarization of *Ptpn22^-/-^* OT-I T cells to a memory-like phenotype prior to ACT enabled prolonged retention of adoptively transferred T cells in vivo following tumor clearance.^47^ In contrast, PTPN22-deletion did not enhance the functional capacity of Her-2-specific CAR T cells against Her-2-expressing tumors or OT-I T cells toward high affinity pOVA-expressing tumors.^105^ These data are largely consistent with findings indicating that the absence of PTPN22 predominantly impacts on T cell responses following weak antigenic stimulation.^25^^,^^26^^,^^48^

A recent study demonstrated that, despite showing enhanced anti-tumor effector capacity, under conditions of chronic, strong antigenic stimulation, *Ptpn22^-/-^* CD8^+^ T cells adopt an exhausted phenotype more rapidly than WT counterparts^33^ (Figure 2). In particular, cotransfer experiments demonstrated that *Ptpn22^-/-^* CD8^+^ T cells initially proliferated more but assumed an exhausted PD-1^+^Slamf6^-^ phenotype and died more rapidly than *Ptpn22^+/+^* cells in ID8-OVA tumor-bearing mice.^33^ The dysfunctional phenotype of adoptively transferred *Ptpn22^-/-^* T cells in these experiments was driven by enhanced IL-2 signaling, resulting from elevated IL-2R expression, and could be partially reversed by PD-1 blockade.^33^ These data indicate that balancing enhanced effector function with a propensity for exhaustion will be key to the future development of PTPN22 as a target for improving anti-cancer ACT therapy (Figure 2). In addition, the lack of enhanced efficacy following deletion of PTPN22 in CAR-T cells suggests that targeting PTPN22 might be most useful in combination with conventional TCR-engineered T cells or tumor infiltrating lymphocyte (TIL)-based ACT therapies.^106^

Several studies have demonstrated that *Ptpn22^-/-^* mice have strong antitumor immune responses following implantation of syngeneic cancer cells.^107^^,^^108^ Thus CD8^+^ T cell responses to MC38 colon carcinoma tumors are elevated in *Ptpn22^-/-^* mice as compared to controls, while PTPN22-deficiency combined with PD-1 checkpoint blockade effectively cleared tumors.^107^^,^^108^ Similarly, growth of B16 melanoma and MC38 tumors was suppressed in PTPN22 R619W knock-in mice compared to controls.^109^ These effects were also associated with greater T cell recruitment to and activation within tumors and decreased numbers of inhibitory myeloid-derived suppressor cells.^109^ Furthermore, analysis of human patient cohorts indicates that expression of the autoimmune-associated *PTPN22* 1858T allele may reduce the incidence of nonmelanoma skin cancers^107^ and be associated with enhanced responses to immune checkpoint inhibitors.^107^^,^^108^ Together these data provide compelling evidence that PTPN22-deficiency or expression of PTPN22 R620W enhances T cell anticancer immunity.

The development of small molecular inhibitors that target phosphatases may represent a therapeutic approach for the treatment of cancer. Several compounds that target phosphatases including PTPN11/SHP-2 and PTPN1/PTPN2 have shown remarkable efficacy in preclinical studies and are in early phase clinical trials for the treatment of cancer.^5^ Importantly, recent studies have shown that PTPN22 is also a druggable target.^9^^,^^108^^,^^110^ Thus, systemic administration of a PTPN22 selective inhibitor, L-1, reduced syngeneic tumor growth in *Ptpn22^+/+^,* but not *Ptpn22^-/-^*, mice and synergized with PD-1 blockade to mediate tumor clearance.^108^ The protective effect of L-1 was mediated via and dependent upon modulation of CD8^+^ T cell and macrophage function. These data indicate that PTPN22 inhibition may represent a viable approach to enhancing cancer immunotherapy.

## Concluding Remarks

PTPN22 has attracted a great deal of interest over the past two decades due to the strong association of SNPs with autoimmune disease and its role as a key regulator of T cell and other leukocyte responses. As documented here, in recent years, the use of mouse models and newer gene-editing approaches in T cells have begun to reconcile some of the controversies regarding the precise impact of disease-associated *PTPN22* polymorphisms on T cell function. The role for PTPN22 in regulating T cell immune responses to infection have been less well studied, whilst recent data indicating a role for PTPN22 in modulating cancer immunity deserve further investigation. Ultimately, it is to be hoped that future studies of PTPN22 function will continue to provide insight into the mechanisms underpinning the regulation of T cell signaling and activation, susceptibility to and pathogenesis of autoimmune disease as well as identifying novel therapeutic approaches for cancer immunotherapy.

## Data Availability

Data sharing not applicable, no new data generated.
